# Discordance of the Urinary and Pleural Fluid Antigen Test and False Positive for Streptococcus pneumoniae in Empyema Secondary to Necrotizing Bacterial Pneumonia

**DOI:** 10.7759/cureus.37458

**Published:** 2023-04-11

**Authors:** David O Shumway, Kevin Kriege, Stuart T Wood

**Affiliations:** 1 Internal Medicine, Keesler Medical Center, Biloxi, USA; 2 Infectious Disease, Keesler Medical Center, Biloxi, USA

**Keywords:** chatgpt, pneumococcal pneumonia, rapid antigen detection test, bronchopulmonary fistula, pleural effusion, empyema

## Abstract

Empyema is a severe complication of pneumonia with high morbidity and mortality rates. Rapid diagnosis and tailoring of antibiotic therapy are crucial to treatment success for these severe bacterial lung infections. A Streptococcus pneumoniae (*S. pneumonia)* antigen test drawn from the pleural fluid rather than a urine sample has been found to have equivalent diagnostic utility to the urinary antigen test. Discordance between these tests is rare.

We report a case of a 69-year-old female with CT imaging findings consistent with empyema and a bronchopulmonary fistula. A rapid *S. pneumonia* antigen test was negative from the urinary sample but positive when drawn from a patient's pleural fluid sample. Final pleural fluid cultures resulted in *Streptococcus constellatus *(S. constellatus). This case demonstrates discordance between the results of urinary and pleural fluid *S. pneumoniae *antigen tests, representing a potential pitfall in using rapid antigen testing on pleural fluid samples. False positives for the *S.* *pneumoniae* antigen in patients with viridans streptococci infections have been documented due to the cross-reactivity of cell wall proteins in different streptococcal species. Physicians encountering bacterial pneumonia of unknown etiology complicated by empyema should understand the potential for discordance and false positives using this diagnostic method.

## Introduction

Empyema, a severe complication of bacterial pneumonia, carries high rates of morbidity and mortality, where approximately 30% of patients require surgical intervention with drainage of the pleural space, and 15% of patients ultimately perish [1]. Pleural fluid analysis is the gold standard in diagnosing empyema and the most important predictor of clinical outcomes. According to the American Association for Thoracic Surgery, it remains a class one recommendation to investigate and sample any pleural effusion discovered in patients with pneumonia or unexplained sepsis with failure to respond to appropriate antibiotic therapy [1]. The presence of purulence, positive Gram stain, or culture from the pleural fluid sample confirms the diagnosis of empyema [2]. Identifying a microbial etiology via culture can be slow and non-specific [3,4]. In >60% of empyema cases, plural fluid cultures fail to yield a pathogen [5,6].

Rapid antigen tests offer the potential for fast and accurate diagnosis of common causative organisms implicated in empyema. In particular, Streptococcus pneumoniae (S. pneumoniae) rapid urine antigen tests demonstrate more sensitivity than blood cultures. Notably, using an S. pneumoniae antigen test drawn directly from the pleural fluid is equivalent to or enhances the diagnostic utility of the urinary antigen test alone [7,8]. Discordance between these tests is rare.

This case report was previously presented as a poster at the 2021 ACP Abstract Day Meeting at the University of Mississippi Medical Center on October 28th, 2021. Additionally, this case report was prepared using assistance from ChatGPT. However, no direct composition was provided by ChatGPT except where explicitly cited, and sources were reviewed and verified by the authors.

## Case presentation

**Figure 1:**
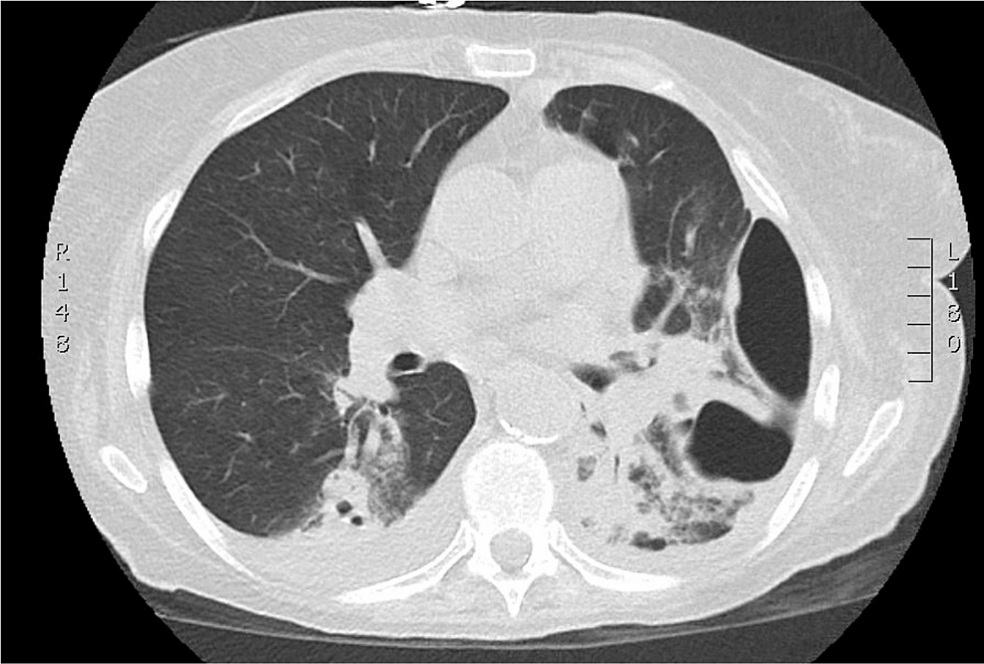
CT chest (axial view) of patient’s necrotizing pneumonia with empyema, complicated by persistent pneumothorax and bronchopulmonary fistula.

**Figure 2:**
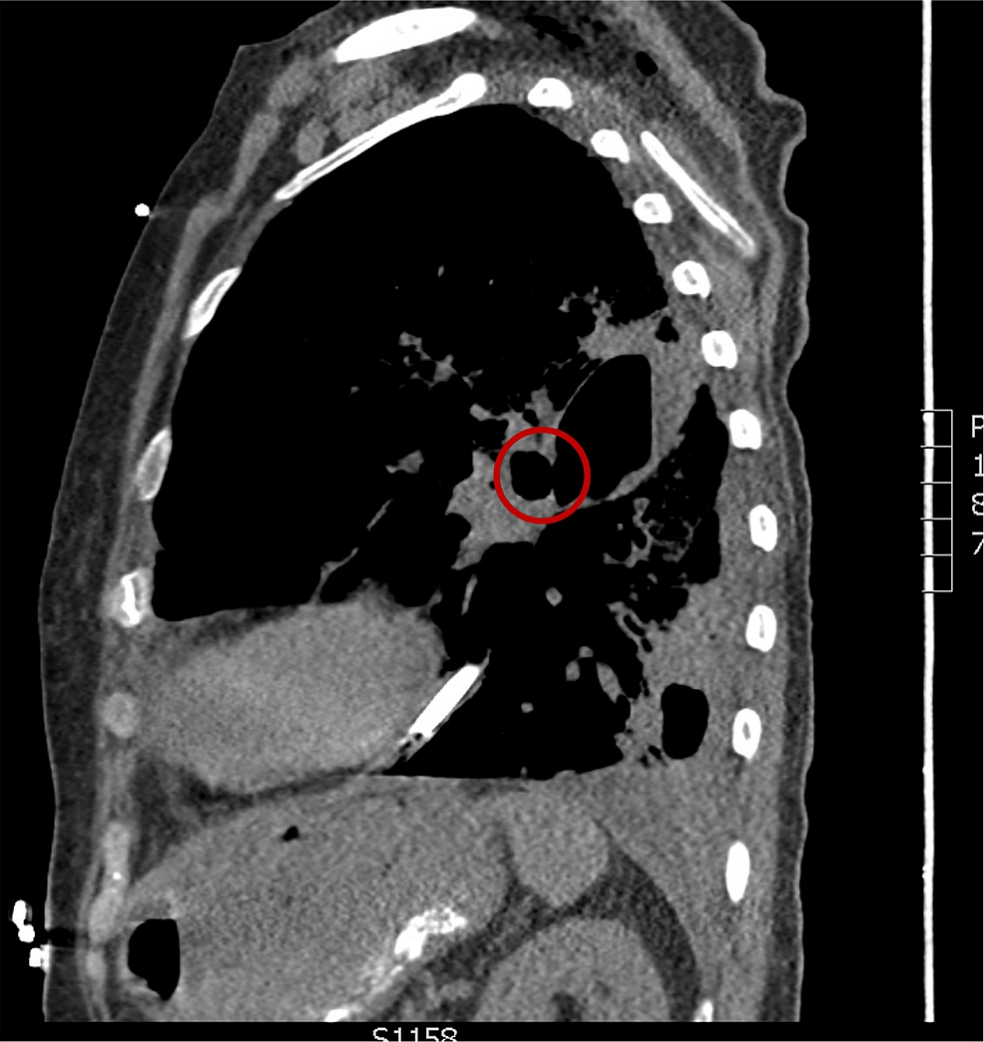
CT chest (sagittal view) showing bronchopulmonary fistula, a cavitary complication of the patient's necrotizing pneumonia

A 69-year-old female with a past medical history of atrial fibrillation, hypertension, tobacco use, and anemia presented to the emergency department with a five-week history of malaise, cough, and progressive shortness of breath. The patient was admitted to the intensive care unit and treated for septic shock secondary to pneumonia. The patient entered atrial fibrillation with a rapid ventricular rate and began to vomit foul-smelling, purulent fluid. The patient quickly became unresponsive, necessitating intubation for emergent airway protection. A left chest tube was placed, returning tan-colored fluid identical to the patient's vomitus. CT imaging revealed findings consistent with empyema and necrotizing pneumonia with a bronchopulmonary fistula on axial (Figure 1) and sagittal (Figure 2) views. The patient had no known incarceration history, Mycobacterium tuberculosis exposure, or travel to endemic countries.

IV vancomycin was started along with IV metronidazole and IV azithromycin. Pleural fluid, urine, blood, and bronchoscopy samples were taken for culture; Gram stain from pleural fluid reflected Gram-positive cocci in chains with Gram-negative rods. The patient was switched to IV vancomycin and IV piperacillin-tazobactam. Metronidazole and azithromycin were discontinued. Methicillin-resistant Staphylococcus aureus (MRSA), PCR, Legionella urine antigen, and Streptococcus urine antigen (Binax-NOW rapid Streptococcal antigen test) resulted as negative; however, Streptococcus urine antigen test was positive when tested from a sample of pleural fluid taken from a chest tube directly. Pleural fluid cultures eventually grew as S. constellatus, a member of the viridans streptococci group, confirmed with VITEK 2 Compact 30 microbial identification system (BioMerieux, USA). The patient was transitioned to moxifloxacin by mouth daily with a plan to complete four to six weeks of therapy and transferred to a site with cardiothoracic surgery capability.

## Discussion

This case demonstrates discordance between the results of urinary and pleural fluid rapid antigen tests and a false positive for S. pneumoniae, a potential pitfall in using rapid antigen assays on pleural fluid samples in empyema.

Understanding the epidemiology of empyema is critical, as initial treatment is often performed empirically [1,9]. S. pneumoniae is responsible for most cases of bacterial community-acquired pneumonia but is less frequently implicated as a single agent etiology of empyema [10-12]. In contrast, viridans streptococci species rarely cause pneumonia but are more common in empyema than S. pneumoniae [5,13]. Viridans streptococci are facultative anaerobes commonly found in the oral cavity [7]. While most often commensal organisms, streptococci in this group have evolved mechanisms of gene exchange, immune evasion [14], and an exceptional ability to form biofilms [7] that can result in severe and difficult-to-treat infections. Treatment and antibiotic sensitivities can vary significantly from species to species within the viridans group and even more when compared to S. pneumoniae isolates; therefore, prompt recognition of the causative organism for tailoring antibiotic therapy is critical for treatment success [9,12].

As stated, rapid tests have been increasingly used as a fast, reliable answer to this clinical problem. The Binax-NOW rapid antigen test used in our case is an immunochromatographic test to detect S. pneumoniae membrane peptidoglycan C-polysaccharide in urine or cerebral spinal fluid [7]. This rapid antigen test carries a high sensitivity (76.9-86.5%) and specificity (84.2-89.7%) in urinary samples [15], yielding an overall high degree of diagnostic accuracy for an exceptionally speedy test. Building on the data from rapid urinary antigen tests, antigen tests drawn from pleural fluid have also been more sensitive and specific than blood or pleural fluid cultures (93.3% sensitivity, 70.6% specificity) [8]. Despite this, false positives for the S. pneumoniae antigen have been documented in patients with viridans streptococcal and certain anaerobic infections [7,8]. The likely mechanism is due to the cross-reactivity of similar cell walls and membrane proteins in different streptococcal species [6].

Discordance between urinary and pleural fluid favors pleural fluid results when false positives occur. Patients often show a negative urinary antigen test and a positive pleural fluid antigen test confirmed by positive S. pneumoniae cultures [6,7]. In the setting of parapneumonic pleural effusion, as shown in our case, a possible explanation for this is expected higher concentrations of antigen (S. pneumoniae peptidoglycan C-polysaccharide) at the source of the infection when compared to more remote sites like peripheral. To our knowledge, discordance with a false positive pleural fluid result and a negative urinary antigen test, proven by failure to grow S. pneumoniae on cultures, has yet to be previously shown in the literature. In our case, the actual causative organism (S. constellatus) was a member of the viridans group and likely exhibited the same cross-reactivity previously described.

## Conclusions

Pleural fluid rapid antigen testing represents a promising method to diagnose Streptococcus pneumoniae infection with a negative urinary antigen test or to confirm a positive urinary antigen test. However, physicians encountering bacterial pneumonia of unknown etiology complicated by empyema and utilizing this novel diagnostic method should understand the potential for discordance and false positives.

## Figures and Tables

**Figure 3:**
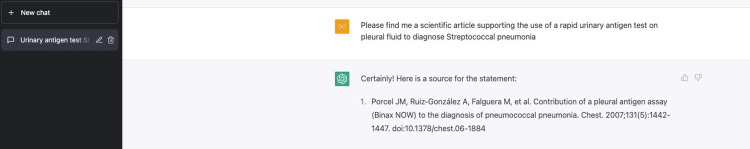
ChatGPT literature review and citation finding

**Figure 4:**
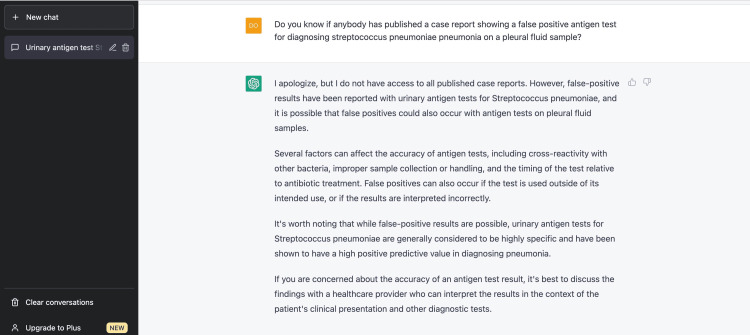
Another example of ChatGPT literature review and intelligent search prompts

## Appendices

ChatGPT is one member of an emerging wave of artificial intelligence technologies and offers promising applications in research and medicine. In the authors' opinion, ChatGPT reliably performed a literature review, citation assistance, and proofreading up to a level qualitatively similar to a human undergraduate or medical student during the preparation of this article. The authors are excited to see where the future of these technologies evolves. Two examples of our experience using ChatGPT (Figures 3, 4) for this article are documented below.
